# Combining Nanopore and Illumina Sequencing Permits Detailed Analysis of Insertion Mutations and Structural Variations Produced by PEG-Mediated Transformation in *Ostreococcus tauri*

**DOI:** 10.3390/cells10030664

**Published:** 2021-03-17

**Authors:** Julie Thomy, Frederic Sanchez, Marta Gut, Fernando Cruz, Tyler Alioto, Gwenael Piganeau, Nigel Grimsley, Sheree Yau

**Affiliations:** 1 Sorbonne Université, CNRS, UMR 7232 Biologie Intégrative des Organismes Marins (BIOM), Observatoire Océanologique, F-66650 Banyuls-sur-Mer, France; thomy@obs-banyuls.fr (J.T.); sanchezf@obs-banyuls.fr (F.S.); gwenael.piganeau@obs-banyuls.fr (G.P.); 2 CNAG-CRG, Centre for Genomic Regulation (CRG), Barcelona Institute of Science and Technology (BIST), Baldiri i Reixac 4, 08028 Barcelona, Spain; marta.gut@cnag.crg.eu (M.G.); fernando.cruz@cnag.crg.eu (F.C.); tyler.alioto@cnag.crg.eu (T.A.); 3 Universitat Pompeu Fabra (UPF), 08003 Barcelona, Spain

**Keywords:** Mamiellophyceae, Chlorophyta, polyethylene glycol, copy number, random insertional mutagenesis, unknown sequences, structural variations, DNA repair, NHEJ, NGS

## Abstract

*Ostreococcus tauri* is a simple unicellular green alga representing an ecologically important group of phytoplankton in oceans worldwide. Modern molecular techniques must be developed in order to understand the mechanisms that permit adaptation of microalgae to their environment. We present for the first time in *O. tauri* a detailed characterization of individual genomic integration events of foreign DNA of plasmid origin after PEG-mediated transformation. Vector integration occurred randomly at a single locus in the genome and mainly as a single copy. Thus, we confirmed the utility of this technique for insertional mutagenesis. While the mechanism of double-stranded DNA repair in the *O. tauri* model remains to be elucidated, we clearly demonstrate by genome resequencing that the integration of the vector leads to frequent structural variations (deletions/insertions and duplications) and some chromosomal rearrangements in the genome at the insertion loci. Furthermore, we often observed variations in the vector sequence itself. From these observations, we speculate that a nonhomologous end-joining-like mechanism is employed during random insertion events, as described in plants and other freshwater algal models. PEG-mediated transformation is therefore a promising molecular biology tool, not only for functional genomic studies, but also for biotechnological research in this ecologically important marine alga.

## 1. Introduction

In most living organisms, a high proportion of genes have unknown functions, and the determination of these functions is a laborious task. To facilitate this functional annotation, different genetic tools are available, such as radiation or chemical mutagenesis [1], genetic engineering [2], RNA interference [3,4] and gene-expression systems [5]. Beyond the advantages of molecular biology providing new methods, these genetic tools allow us to increase the precision of analyses, the throughput and the diversity of organisms that it is possible to study. Random insertional mutagenesis, which entails the integration of exogenous DNA (typically transposable elements, plasmids or viruses) into the genome, is one of the most powerful genetic tools to disturb gene function. This has emerged as the method of choice to produce random genome-wide mutant collections in cultivable organisms with short life cycles that are amenable to genetic transformation [6]. Different methods have been developed for nuclear transformation in many model organisms to enable functional genetic characterization, but there is now growing recognition of the need to study a larger number of “non-model” or “emerging model” systems [7].

In unicellular algae, for many years, the development and application of molecular genetic approaches have been largely confined to the freshwater model alga, *Chlamydomonas reinhardtii* [8]. Microalgae span an immense breadth of taxonomic and genetic diversity, and although they offer opportunities for biotechnology, they are poorly exploited among microorganisms for genetic engineering [9]. The need to develop marine model organisms has been recognized by the Environmental Model Systems (EMS) Project, a multiteam collaboration aiming to advance DNA-delivery protocols for marine protists. Prior to the EMS project, only a handful of marine protists were genetically transformable, and this figure has increased to ~20 [7]. The continued development of genetic manipulation of marine algae opens the door to realizing high-throughput functional studies and development of strains to synthesize high-value products, such as biofuels, food additives, pigments and pharmaceuticals.

The marine green alga *Ostreococcus tauri* (class Mamiellophyceae) is the smallest free-living eukaryote known [10] and a representative of an ecologically important group of marine phytoplankton [11]. Its ease of growth in the laboratory, simple cell organization (one chloroplast, one mitochondrion, a single Golgi body, no cell wall), and its small haploid compact genome (8000 genes, 13 Mbp) [12], makes it an attractive model marine alga. *O. tauri* was shown to stably integrate an overexpression plasmid [13] and was amenable to targeted insertion by homologous recombination by electroporation-mediated transformation [14]. Recently, a simplified method to transform *O. tauri* using polyethylene glycol (PEG) has been described [15]. This approach was first presented in the tobacco plant [16], and in yeast and bacterial models [17,18,19], as well as in protoplasts [20,21,22]. In algae, the first optimization of PEG transformation was reported in the unicellular red alga *Cyanidioschyzon merolae* [23,24]. Overall, PEG-mediated transformation has shown many advantages, including being simple and fast to perform, with a high transformation efficiency. However, genomic characterization of how exogenous DNA is integrated using this method, such as determination of the insertion site and copy number has yet to be explored. Currently, the genomic characterization of transformation in algae has been largely limited to *Chlamydomonas*. Molecular approaches that allowed the copy number of the vector to be determined and the genomic sequences flanking the foreign DNA to be described have been based on the hybridization of radioactive DNA fragments [25], the plasmid rescue technique [26], thermal asymmetric interlaced polymerase chain reaction (PCR) [27,28], restriction-enzyme digestion combined with PCR [29,30], the adaptator-linked PCR [31], hairpin-PCR [32] or *Chlamydomonas* MmeI-based insertion site sequencing (ChlaMmeSeq) [33].

Nonetheless, most of these molecular tools are labor-intensive and provide relatively low-resolution information on the DNA insertion events. Neither can they detect the presence of small insertion or deletions (indels), duplications and chromosomal rearrangements, often observed at the junction site of transfer DNA (T-DNA) integration in *Agrobacterium*-mediated plant transformation [34,35]. The development of high-throughput next-generation sequencing (NGS) technologies has enabled significant progress by quickly providing complete high-resolution genomes at relatively low cost [36]. NGS has proven feasible as a complementary or alternative method for the characterization of transgene insertion events, having been applied in model plants and animals [37,38] where the presence of DNA structural variations at the insertion site have also been observed [39,40].

Here, we report the use of Illumina resequencing combined with Oxford Nanopore technology (ONT) sequencing to produce *de novo* genome assemblies of five independent clonal transformant lines of *Ostreococcus tauri* strain RCC1115 in order to characterize insertional events. These data demonstrate that PEG-mediated transformation is a robust molecular tool leading to the integration of a single or few copies of transforming DNA that is stably maintained in the algal genome. Moreover, we provide a reliable demonstration of the existence of frequent structural variations at the insertion locus, which suggests that *O. tauri* uses a nonhomologous repair mechanism for the introduction of foreign DNA into its genome.

## 2. Materials and Methods

### 2.1. Algal and Viral Culture Conditions

All progeny clonal lines (around 1200 transformed and 200 untransformed clonal lines) derived from *Ostreococcus tauri* RCC1115 (Roscoff Culture Collection) strain were maintained in liquid L1 medium (NCMA, Bigelow Laboratory for Ocean Sciences, USA) made with autoclaved offshore seawater (MOLA station: 42° 27′ 11″ N, 3° 8′ 42″ E), diluted with Milli-Q water to give a final salinity of 30 g L^−1^, and filter-sterilized through 0.22 µm filters. Cultures were kept under a photoperiod cycle of 12 h:12 h light:dark (50 µmol photon m^−2^ s^−1^ white light) at 15 °C, unless otherwise specified.

Cell densities were counted using a Beckman–Coulter Cytoflex flow cytometer (excitation wavelength laser 488 nm) by chlorophyll autofluorescence for algae (detection filter > 620 nm) and by SYBR Green I fluorescence for bacteria (detection bandwidth 525–540 nm channel). Cells were fixed using glutaraldehyde (0.25% final concentration) and pluronic acid (0.10% final concentration) for 15 min in the dark, then SYBR Green I (Ozyme,France, ref LON50512) was added for 15 min to stain bacteria.

A selection of 20 viruses specific to *Ostreococcus tauri* with a range of host strain specificities as described in [41] were grown in liquid culture under the same conditions as previously described. Viral lysates obtained three days post‑inoculation were filtered through filters with a 0.22 µm pore size, then stored at 4 °C for use in virus specificity tests that were performed using the plaque assay described in [41].

### 2.2. Transformation and Screening for Host Susceptibility or Resistance to Prasinoviruses

Insertional mutagenesis was performed by transformation using polyethylene glycol (PEG), as described in [15]. For the transformation experiments in this study, 50 mL of culture at a density of 50 × 10^6^ cells·mL^−1^ was used, in order to obtain at least 2000 transformed clones. The culture was centrifuged for 10 min at 6000× *g* at 20 °C, and the pellet was resuspended and gently mixed with 500 µL PEG 4000 (30% PEG final concentration), and 2 µg carrier tRNA. Then, 10 µg of ScaI-digested pOLK4 DNA [15] was added, followed by incubation of the mixture for 2 min at 20 °C. The cells were then diluted into 40 mL of fresh L1 medium, and transferred to a growth chamber for 6 h to maximize the frequency of transformation. As a control, a cell suspension without PEG treatment and without the addition of DNA was prepared in parallel, described here as the untransformed clonal lines. Finally, both transformed and untransformed control-cell suspensions (1 mL volume final) were mixed with a solution of 2.1% (*w*/*v*) low-melting-point agarose (1 mL) in sterile Milli-Q (Merck, Darmstadt, Germany) water (maintained at 60 °C in a water bath) and 8 mL of L1 medium at 20 °C with 2 mg·mL^−1^ G418 (Sigma-Aldrich, MO, USA, ref A1720) or without antibiotic, respectively, and poured into Petri dishes (diameter 55 mm). The Petri dishes were transferred into a culture chamber at 20 °C with 100% humidity and incubated until colonies appeared, usually for at least 3 weeks. Individual *O. tauri* colonies were picked from the semisolid medium with a sterile pipette tip and transferred into 200 µL liquid L1 medium in 96-well plates. Over 1200 transformed clones were screened for by antibiotic (G418) selection and 200 untransformed clones were collected. Second, a phenotypic screen for susceptibility or resistance to the virus strain OtV09-578, to which the parental RCC1115 is susceptible, was carried out on all clonal lines (untransformed and transformed). The phenotypic screening was performed in liquid L1 medium in 96-well plates. Control uninfected cultures were mock-inoculated with 50 µL of L1 medium, while a concentrated viral suspension of 50 µL was added to the test cell cultures to a final volume of 200 µL. The dynamics of infection were followed under the growth conditions described above by visual inspection of the green coloration of the culture over 3 weeks. Cultures were considered virus-susceptible if visible loss of green color occurred (indicative of viral lysis of cells) and as potentially virus-resistant candidates if there was no visible color change compared to the uninfected controls.

### 2.3. Illumina and Oxford Nanopore Technologies (ONT) Sequencing

Seven clonal lines, including two untransformed controls and five transformed lines, (Table 1) were selected for genome sequencing. Genomic DNA for each clonal line was extracted with the CTAB (cetyltrimethylammonium bromide) protocol, as previously described [42]. DNA quality was evaluated by absorbance at both 260/230 nm and 260/280 nm ratios in a Nanodrop and confirmed by electrophoresis and visualization in a 0.8% agarose gel. DNA was quantified in 1 µL by fluorimetry (Quantus™, Promega, Lyon, France) using a QuantiFluor^®^ dsDNA system kit (Promega, Lyon, France, ref E2670).

The short-insert paired-end libraries (2 × 101 bp) for whole genome sequencing (Illumina Hiseq 3000/4000 system) were prepared with a KAPA HyperPrep kit (Roche, Bâle, Switzerland) with some modifications. As a function of the material availability, 0.07–1.0 µg of genomic DNA was sheared on a Covaris™ LE220-Plus (Covaris, Woburn, MA, USA). The fragmented DNA was size-selected with AMPure XP beads (Agencourt, Beckman Coulter), end-repaired, adenylated and Illumina-platform-compatible adaptors with unique dual indexes and unique molecular identifiers (Integrated DNA Technologies, Coralville, IA, USA) were ligated. Depending on the library concentration, the adaptor-modified end library was enriched by 4–15 PCR cycles, except for libraries with starting material of ≥1.0 µg, which were further processed without PCR amplification. The libraries were quality-controlled on an Agilent 2100 Bioanalyzer with the DNA 7500 assay (Agilent, Santa Clara, CA, USA) and quantified by Kapa Library Quantification Kit for Illumina platforms (Roche, Bâle, Switzerland).

ONT sequencing was performed on the seven transformed lines (Table 1). The 1D Genomic libraries for the long-read Nanopore sequencing were prepared using Native Barcoding Expansion 1–12 and 13–24 kits (ONT) and Ligation sequencing kit SQK-LSK109 (ONT) following the manufacturer’s recommendations. A total of 2.5 µg of genomic DNA was used in the DNA repair (NEBNext FFPE DNA Repair Mix) and end-repair reactions (NEBNext Ultra II End repair/dA-tailing module reagents) (New England Biolabs, NEB, Ipswich, MA, USA), followed by native barcode ligation with Blunt/TA Ligase Master Mix (NEB) and Native Barcoding Expansion 1–12 and 13–24 kits. After ligation of the barcodes, the samples were combined into an equimolar pool of 12 or 13 samples, and adapter ligation reaction was carried out using Adapter Mix II (ONT) together with the NEBNext quick ligation reaction buffer 5X (NEB) and Quick T4 DNA Ligase (NEB). The library was purified with 0.4X AMPure XP Beads (Beckman Coulter, Brea, CA, USA), washed with short fragment buffer (ONT) and eluted with Elution Buffer (ONT). Nine sequencing runs were performed using R9.4.1 flow cells (ONT) on the GridION instrument (ONT). The MinKNOW GUI (v3.5.4) interface QC (ONT) was run in order to assess the flow-cell quality, and this was followed by the flow-cell priming. After flow cell priming, the presequencing mix comprising sequencing buffer and loading beads (ONT) were combined with the final library and loaded in the flow cell. The sequencing data were collected over 48 h and the quality parameters of the sequencing runs were further monitored by the MinKNOW platform. The raw data were base called with Guppy 3.2.6.

### 2.4. Long-Read Assembly

The sequencing data from each of the five transformed clonal lines (Table 1) were used to produce a long-read assembly (using ONT reads only) and a hybrid assembly (using both Illumina and ONT reads). The long-read assemblies were obtained with Flye version 2.6 [43] with a minimum overlap between reads of 1 Kb (-m 1000), 2 polishing iterations (-i 2) and genome size 14 megabases (-g 14m). In addition, we obtained a hybrid genome assembly with MaSuRCA v3.4.1 [44,45] using raw Illumina paired-end libraries (2 × 101 bp, 517–837x) and the raw ONT reads (N50 7.4–13.4 Kb and 42–96x) to construct mega-reads and assembled them with Flye version 2.5.

### 2.5. Analysis of Insertion Sites Using Illumina Short-Read Sequencing

The quality of raw reads was checked using FastQC (v0.11.7). For each clonal line, the reads were mapped with the Burrows–Wheeler Aligner (BWA v0.7.17) using maximum exact matches (BWA-MEM) and a minimum seed length of 20 while keeping the other default parameters [46]. Reads were mapped to the chromosomal sequences the *Ostreococcus tauri* RCC1115 (v1.0) reference genome (BioProject number on NCBI: PRJNA337288), including the chloroplast and mitochondrial genomes (see Table S1 for the GenBank contig identifiers and accession numbers), as well as the 6Kbp pOLK4 vector sequence. Throughout this study, the chromosome numbers we reported for RCC1115 were the equivalent chromosome numbers to the genome of *O. tauri* strain RCC4221 [47], as whole-genome comparisons have revealed a low level of polymorphism (Φ = 0.01) between these two strains [48] (Table S1). Mapping results were visualized in the Integrative Genomic Viewer (IGV v2.4.16) implemented with the reference genome annotation available from the JGI website accessed on 15 March 2020 (https://phycocosm.jgi.doe.gov/Ostta1115_2/Ostta1115_2.home.html). To localize the insertion site, first all unmapped reads and mates were removed with Samtools (options: -view -F 12) software (v1.10), then reads that spanned the junction between the chromosome and vector sequence were kept by manual methods, i.e., reads that spanned the junction were manually parsed from the alignment file (in .sam format) by text-string searches for the vector identifier.

### 2.6. Analysis of Insertion Sites in ONT Long-Read Assemblies

Analyses were carried out on assemblies from both assembly methods (Flye and Masurca), but only the results from the hybrid assembly are presented in view of its better assembly quality at the integration site, which was confirmed by PCR resequencing (see Section 2.7). Assembly quality was also assessed by remapping the Illumina reads against the *de novo* ONT contigs using BWA-MEM (v0.7.17) (options: –k 20). The visualization and the analysis of assemblies was performed using Geneious software (v11.0.3+7). The contig containing the vector’s sequence was identified by a BLASTn (v2.10.1+) search using the pOLK4 sequence as a query (accepting the top high-scoring pair with e-value < 1e−5 and at least 95% identity). The integration of the vector into the chromosome was visualized by dot plot using Mummer (v4.0.0).

### 2.7. Confirmation of Structural Variations Flanking the Insertion Using PCR

PCRs were performed on transformants in which small indels flanking the insertion sites had been detected in the *de novo* hybrid assemblies (T3, T6 and T16). Two sets of primers for each sample were designed to amplify the 5′-end and the 3′-end junctions of each insertion site (Figure S7). PCRs were conducted as follows: 2 µL of extracted DNA was added to a 48 µL reaction containing 1X PCR buffer, 1 mM MgCl_2_, 0.2 mM deoxyribonucleoside triphosphate (dNTP), 0.5 µM of each primer, 0.1 mg·mL^−1^ BSA and 1.25 U of *Taq* DNA polymerase (GOTaq^®^ G2 Flexi M7805, Promega), which was amplified in a Mastercycler nexus system (Eppendorf) under thermocycling conditions comprising initial denaturation at 94 °C (3 min), 40 rounds of denaturation at 94 °C (30 s), annealing at 56 °C (30 s), extension at 72 °C (30 s) and final extension at 72 °C (4 min). PCR products were run on a 0.8% agarose electrophoresis gel in 0.5% TAE (Tris-acetate-EDTA) buffer and visualized under ultraviolet light after ethidium bromide staining. PCR products were purified using a Wizard^®^ SV gel and PCR clean-up system kit (Promega, ref A9281) and then sequenced by Sanger sequencing technology. Sequences were cleaned and analyzed with Geneious^®^ software (version 2.1).

### 2.8. Bioluminescence Assay

The cellular concentration for each culture was adjusted to 20 × 10^6^ cells·mL^−1^ in a final volume of 200 µL in each well of a 96-well white plate (Greiner Bio-OneTM LUMITRAC, SAS, Les Ulis, France), then D-luciferin (Pierce, Waltham, MA, USA, ref 88293) was added to a final concentration of 100 µM. The plate was placed at room temperature in the dark for 10 min before measuring luminescence with a Victor Nivo 3F Microplate Reader Spectrofluorimeter (PerkinElmer Reader, Waltham, MA, USA, Ref: 396532, Dominique Dutscher) by digital photon counting. A bar plot was constructed using the ggplot2 data visualization package implemented within the RStudio environment.

### 2.9. Data Availability

The parental *O. tauri* strain RCC1115 used for transformation and the seven clonal lines whose complete genomes have been sequenced in this study have been deposited in the Roscoff Culture Collection (http://roscoff-culture-collection.org/ accessed on 15 March 2021) with the following strain numbers: T3 (RCC7079), T6 (RCC7080), T12 (RCC7081), T14 (RCC7082), T16 (RCC7083), NT1 (RCC7084) and NT10 (RCC7085). The Illumina–ONT hybrid assemblies and raw Illumina and ONT reads of the *O. tauri* lines analyzed in this study are available from the European Nucleotide Archive (ENA) under project accession number PRJEB43294. The sequence of the pOLK4 vector is available under GenBank accession number MW598458.

## 3. Results

### 3.1. Illumina Paired-End Sequencing Data and Mapping to Wild-Type and Transformed Lines

High-throughput sequencing yielded a total of 90 × 10^6^ and 98 × 10^6^ paired-end reads for untransformed clonal lines NT1 and NT10, respectively, and 122 × 10^6^, 75 × 10^6^, 86 × 10^6^, 79 × 10^6^ and 100 × 10^6^ for the transformed lines T3, T6, T12, T14 and T16, respectively. By mapping paired-end reads to the reference *O. tauri* RCC1115 v1.0 nuclear and organellar genomes (14.8 Mbp), we obtained between 500 and 800× average coverage of the nuclear genome (Table 1). The coverage for each chromosome was similar to the average coverage of the nuclear genome, whereas the coverage of the mitochondrial and chloroplast genomes was 4-fold more than the nuclear genome (Table S1), indicating there were four organelle genome copies per cell.

### 3.2. The Integrity of the Transgene in Each Line Is Variable

**Figure 1:**
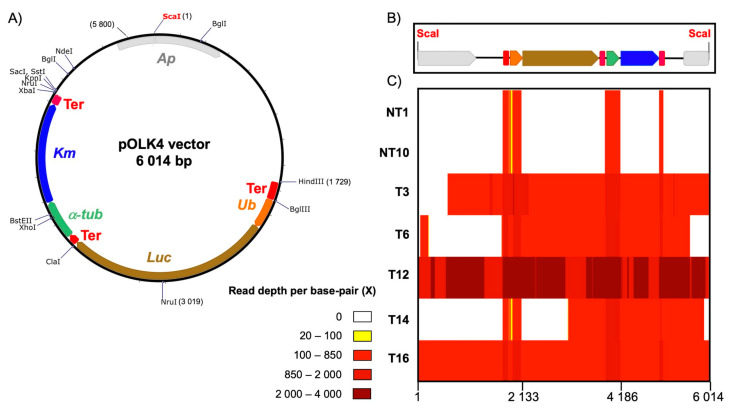
Sequence map and read coverage of the pOLK4 vector. (**A**) Map of the circular pOLK4 vector. Gray arrow: Ap, ampicillin-resistance gene; blue arrow: Km, kanamycin (G418) antibiotic-resistance gene used as a selectable marker for algal transformation; brown arrow: Luc, luciferase used as a luminescence reporter gene; orange arrow: ubiquitin promoter; green arrow: α-tubulin promoter; and red block: Ter, α-tubulin terminator. Positions of restriction sites are shown. (**B**) Map of the linear pOLK4 vector that the algal cells were transformed with highlighting of the Sca-I restriction site (red) used to linearize vector DNA. Colors of the gene maps are as in (**A**). (**C**) Heatmap of short reads from untransformed (NTx) and transformed (Tx) lines mapped against the linearized vector, showing no read coverage (white) to high read coverage (dark red).

To determine the integrity of the transformed DNA, we measured the coverage of the Illumina read pairs that mapped to the whole pOLK4 vector (Figure 1C). Note that the pOLK4 construct used for this study contained sequences from *O. tauri* that were expected to recruit genomic reads (Figure 1): a ubiquitin promoter sequence found on RCC1115 chromosome 12 (position 173,766–175,000 bp), as well as α-tubulin promoter and terminator sequences (position 119,519–120,967 bp) from the same chromosome [15]. Reads from both NT1 and NT10 untransformed lines mapped only to the regions of the vector-encoding promoter and terminator sequences, confirming the control lines did not contain the vector sequence. Reads from the transformed lines T3, T12 and T16 mapped to more than 90% of the vector sequence length, while reads from T6 and T14 covered less than 75%. This showed the integrity of the transformed vector was variable; in some lines it was complete, whereas in others, portions of the vector sequence were lost. Specifically, deletions occurred mainly at the linearized vector extremities, with the exception of line T6, where a fragment of the vector’s 5′-end containing a part of the ampicillin resistance gene was retained (Figure 1C). Consistent with the selection of transformants on G418 (Km), no deletions occurred in the G418 antibiotic-resistance selectable-marker gene.

### 3.3. Most of the Transformants (4 out of 5) Carried One Copy of the Transforming DNA

To quantify the number of insertions of the vector sequence in each transformed line, we used an NGS strategy to compare the coverage of transformed DNA to the coverage of the nuclear genome. Effective coverage of the pOLK4 vector was 649×, 306×, 1947×, 208× and 531× for the T3, T6, T12, T14 and T16 lines, respectively (Table S2), which was in the same order of magnitude as the average coverage of the nuclear genome (Table 1). However, given the heterogeneous integrity of the pOLK4 vector, to determine the copy number of the transforming DNA as accurately as possible, we calculated the ratio of read coverage of the G418 selectable marker to the coverage of a single-copy housekeeping gene, DNA directed DNA polymerase II, family B (OtaPolB) (Table 2). We estimated that four transformants (T3, T6, T14, T16) harbored one copy of the transforming DNA in their genomes, while T12 carried four copies (Table 2).

### 3.4. Vector Insertions Occurred at a Single Genomic Location in Each Transformant

**Figure 2:**
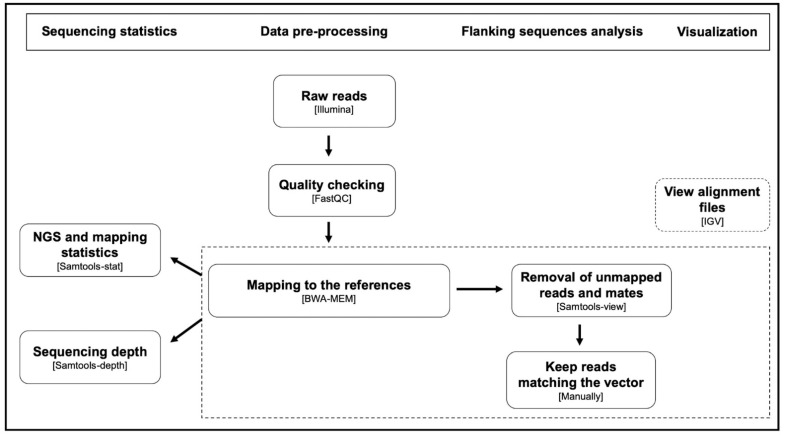
Procedure for finding the insertion sites of transforming DNA in the host genome using short-read NGS data.

The insertion events of the pOLK4 vector were characterized according to the bioinformatic workflow presented in Figure 2, based on the strategies used in genetically modified crops [37], and also as described by Yang et al. in module 1 [49]. First, the quality of the raw read data was checked using the FastQC program (v0.11.5), confirming that the quality was sufficiently high to carry out analyses on the raw reads. Secondly, we mapped the shorts-reads from the transformed lines against the reference genome and the vector sequence with maximal exact matches. Next, we manually retrieved only reads of a pair mapping at one end to the reference genome and at the other end mapping to the vector sequence, which we called “junction pairs”, to locate the vector insertion site. Finally, we visualized the coverage around the predicted insertion site to indicate any local insertions and/or deletions of the genome.

**Figure 3:**
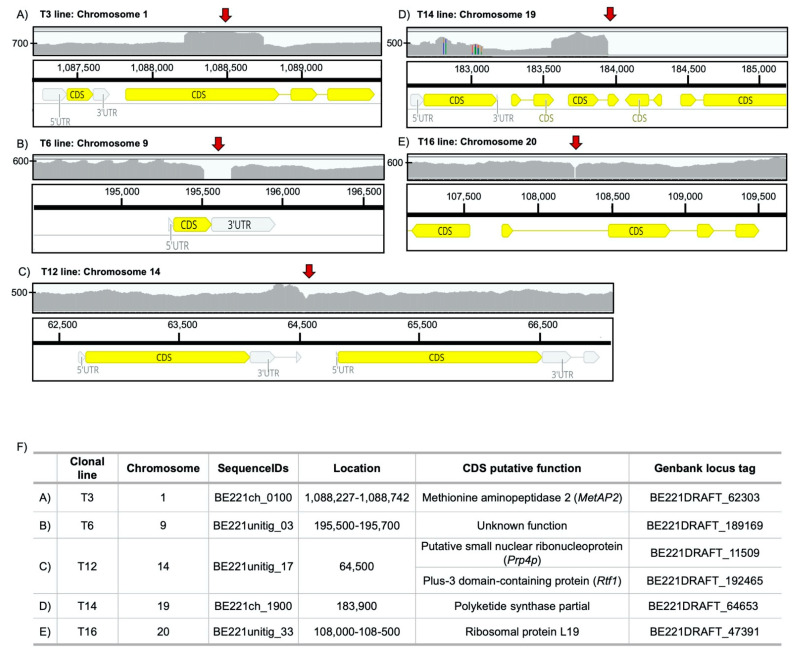
IGV (Integrative Genomics Viewer) visualization of read coverage and identification of insertion sites. Genomic maps of the vector-insertion site in (**A**) T3, (**B**) T6, (**C**) T12, (**D**) T14 and (**E**) T16 transformed clonal lines. The coverage plot is shown in the top panel (*Y*-axis the coverage (X) scale), the locations of insertion sites are shown with red arrows and the gene annotation of the reference genome is shown in the bottom panel. (**F**) Exact location of the sites of vector integration and putative functions of genes surrounding the insertion site.

For lines T3, T6 and T16, the junction pairs on both ends of transforming DNA were detected, enabling the characterization of the insertion site. In line T3, the vector insertion was predicted to be on chromosome 1 in an exonic region of a gene coding for a methionine aminopeptidase 2 (*MetAP2*), where the insertion was flanked by a small duplication of the genomic sequence (Figure 3A,F). The vector insertion site of the T6 lineage was located on chromosome 9, inducing a deletion of about 200 bp from the 3′-extremity and part of the 3′-UTR of a coding sequence with no known function (Figure 3B,F). In line T16, the vector insertion mapped to a single locus on chromosome 20, which also produced a deletion of 10 bp in the intron of ribosomal protein L19 (Figure 3E,F).

In lines T14 and T12, although we could locate the insert, we could not fully determine the structure of the insertion site from this strategy using short-read mapping. For the T14 line, only junction reads spanning the left-hand side of the insertion were found, which were located on chromosome 19 within a partial gene encoding a type I polyketide synthase (*PKS*) (Figure 3D,F). The absence of junction reads to the right-hand side of the insertion site was likely due to deletion of the equivalent region of chromosome 19 in T14, evidenced by the lack of coverage downstream of the insertion (Figure 3D). This chromosome in *O. tauri* is termed the small outlier chromosome (SOC) due to its lower GC content and higher proportions of repeats and species-specific genes than the other chromosomes, but nonetheless is conserved in all the sequenced genomes of Mamiellales [50]. Furthermore, altered transcription and hypervariability in size and genomic content of the SOC are linked to resistance to viruses in *Ostreococcus* species [48,51,52,53]. In keeping with the SOC’s higher propensity for structural variation, it is likely that DNA integration has promoted a deletion of more than 100 kb of the SOC, as the deletion was adjacent to the insertion locus, and short-read mapping in the other six lines did not show similarly large structural variants (Figure S1). We observed a loss of 31 predicted unique genes encoded by the wild type (Table S3), however ~35% of the deleted region comprised repeat sequences located elsewhere on chromosome 19 (Figure S1), including nine presumably redundant genes. Finally, the T12 transformant was estimated to bear four vector copies (Table 2), and it accordingly recruited the highest number of junction pairs, all of which were located on chromosome 14 at a single locus spanning the left-hand end of the insert. This insertion fell in an intergenic region between a small nuclear ribonucleoprotein gene (Prp4p) involved in RNA splicing and Plus-3 domain-containing protein gene (Rtf1) (Figure 3C,F). Rtf1 forms part of the Paf1 complex (Paf1C) that consists of five proteins (Paf1, Rtf1, Cdc73, Ctr9, and Leo1) in eukaryotic systems, where it functions with RNA polymerase II in transcriptional processes and is also associated with RNA polymerase I [54]. Overall, as all junction pairs were located in the same region on chromosome 14 at approximately four times the average read coverage; this suggested multiple insertions occurred at the same locus.

### 3.5. Vector Integration Is Associated with Structural Variation at the Insertion Site

**Figure 4:**
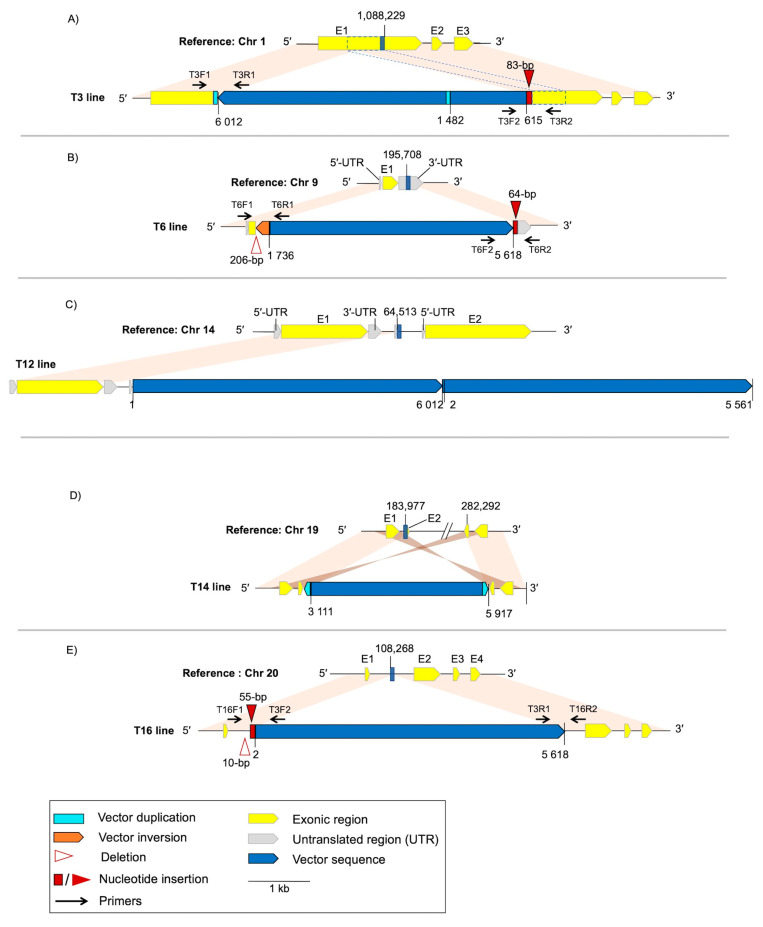
Schematic illustration of the vector integration sites and associated structural variations in contigs assembled from transformants compared to the reference genome. Homologous sequences between the reference and the clonal lines are connected by colored blocks (light orange, same orientation; dark orange, inverse orientation) (**A**) T3 line. (**B**) T6 line. (**C**) T12 line; the entire vector sequence occurs twice in tandem at the end of the contig. (**D**) T14 line. Note that the map of the reference chromosome 19 sequence shows two distant regions separated by a break. (**E**) T16 line. Primers (black arrows) targeting the flanking regions have been designed to confirm the insertion–deletion events (red) in the T3, T6 and T16 lines.

In order to gain insight into the structural changes at the vector integration site, we resequenced all of the transformed clonal lines using ONT and performed *de novo* assemblies with the long reads, as well as hybrid assemblies with both long and short reads. The alignment of the assembled contigs back to the chromosome of the reference strain where the insertion was predicted from the short-read analysis confirmed the exact location of the integration site. Likewise, aligning the contigs containing the insert against the pOLK4 vector (Figure S2) confirmed the variable completeness of the vector sequence that we observed in the Illumina short-read data (Figure 1C). The hybrid assembly enabled the description of structural variations, such as insertions, duplications, deletions and inversions, in both the chromosome and the vector (Figure 4), which could not be resolved from the short-read mapping strategy.

In the T3 line, the insertion of the vector was accompanied by an 83-bp insertion of unknown origin at the vector’s 5′-end and a 62-bp duplication of the noncoding region of the pOLK4 vector sequence (position 1482–1543 bp) at the 3′-end. The inserted sequence was flanked by a 518-bp direct duplication of part of the *MetAP2* gene at the integration site (Figure 4A).

In the T6 line, several variations were observed (Figure 4B): a 206-bp deletion of the 3′-end of the gene at the integration site; a 64-bp insertion of sequence of unknown origin at the vector’s 3′-end; and rearrangement in the vector sequence itself, with a 171-bp inversion corresponding to part of the ampicillin resistance gene (position 40–210 bp) at the vector’s 5′-end.

In the T16 line, we observed a 10-bp deletion of genomic sequence and a 55-bp insertion of viral origin (a partial gene encoding an integrase Genbank protein ID: XP_022839977.1) at the vector’s 5′-end (Figure 4C).

In the T14 line, the integration of the vector was more complex than in the other lines, involving a 90-bp inverted duplication of the part of the ampicillin-resistance gene from the vector’s 3′-end (position: 5828–5917 bp), and the insertion of the vector occurring between two 414-bp inverted repeat sequences of chromosome 19 origin (Figure 4D). In the reference chromosome 19, these 414-bp inverted repeat sequences are separated by ~98 kb, and it is primarily this intervening sequence that has been deleted in T14. We observed the vector integration occurred adjacent to the telomeric sequence at the end of the contig, indicating that the integration locus is located at the chromosome end in T14 and the local structure of the vector insertion was resolved in the assembly. As the region homologous to the vector integration site is not at the chromosome end in the reference (Figure S2), this further indicates a larger rearrangement event occurred within chromosome 19 of T14 that placed this region at the chromosome extremity.

In the T12 line, although four copies of the vector have been estimated from the Illumina short-read coverage, the hybrid assembly showed tandem insertion of the vector in only two copies (Figure 4E). However, as the vector sequences were located at the contig’s 3’-end (Figure S2C), and alignments of ONT long reads against partially polished assemblies showed compressed coverage, the tandem vector repeats likely led to an assembly breakpoint and thus the insertion structure was not completely resolved. From examination of the long-read alignments, it was clear that the vector had been inserted multiple times in tandem with at least one vector copy inserted in the inverse orientation.

To verify the presence of the small indels flanking the vector integration sites, we designed primers to the junctions between the vector and chromosomal sequences (Figure 4) and performed PCR. The amplification products (Figure S5) were subjected to Sanger sequencing that confirmed the sequence of these small indels for T3, T6 and T16 lines (Figure S6) corresponded exactly to the sequence in the hybrid assemblies.

### 3.6. Inserted DNA Is Transcribed in Transformed Lines

**Figure 5:**
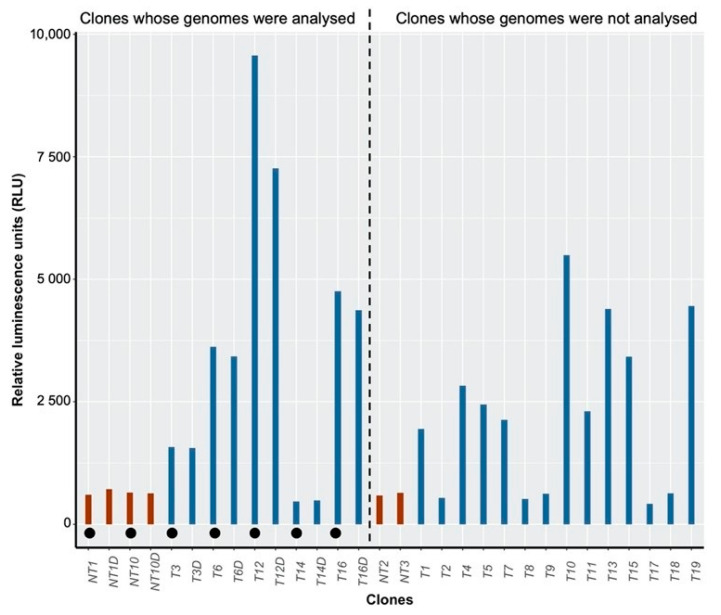
Firefly luciferase assay of transgenic clonal lines. Emission of light measured expressed in relative luminescence units (RLU) from 19 transformed clonal lines (blue bars) and 4 untransformed clonal lines as a control (red bars). The stability of the vector within the host genome was analyzed in the 7 lines studied in this study by measuring the fluorescence emitted from samples (NT1D, NT10D, T3D, T6D, T12D, T14D, T16D) thawed 6 months after transformation. Clonal lines studied in this work are distinguished by filled black circles. Raw data are available in Table S4. Each sample was measured once.

Thousands of clonal lines, both transformed and untransformed, were generated in this study, from which we selected the seven clonal lines whose genomes were sequenced. A selection of these clones was maintained in liquid culture. In order to enlarge our sample size and to have a broader view of the level of expression of the inserted DNA, we included an additional 14 transformed and 2 nontransformed clonal lines to this analysis. The expression of the inserted DNA was measured from the vector’s luciferase reporter across 23 independent clonal lines (Figure 5). Control lines exhibited a low basal-level luminescence, establishing background levels in the absence of luciferase expression. In order to assess the stability of integration of the vector over time, we thawed a subset of seven lines that had been cryopreserved six months earlier (NT1D, NT10D, T3D, T6D, T12D, T14D, T16D) and assayed their luciferase expression in parallel with the 23 lines, which had been maintained by repeated subculture. The expression of luciferase was similar between cryopreserved lines and lines that had not been frozen (Figure 5), indicating vector integration and reporter expression were stable over this six-month timeframe. We observed a high variation in luminescence in transformed lines demonstrating highly variable protein expression (Figure 5). Six out of 19 transformed lines (~32%) showed background luminescence levels in agreement with previous work that found several transformants lacked reporter expression [15]. We observed the absence of luciferase expression for the T14 line, which is consistent with the deletion of the reporter gene in this line that we determined in the NGS analysis. This result suggests that the lack of luciferase expression previously observed was similarly due to deletion of the reporter gene. We established that luciferase activity was not related solely to the number of copies of the vector, as evidenced by the range of luminescence in the T3, T6 and T16 lines, each of which contained a single copy of the vector. In these lines, the variation in luminescence was likely due to variation in gene regulation specific to the genomic context of the integrated vector. Nonetheless, the T12 line showed the highest luciferase activity and also had the highest number of vector copies, as determined by the genome resequencing, which provides some evidence that vector copy number also contributed substantially to reporter expression levels.

## 4. Discussion

### 4.1. What Could Be the Mechanism Underlying the Random Insertion of DNA by PEG-Mediated Transformation?

External mutagenic agents, such as ultraviolet (UV) radiation or ionizing radiation (IR), which are naturally present in the environment [55], can severely damage eukaryotic cells. The DNA lesions are detected by DNA damage-response (DDR) pathways, which include direct repair, base excision repair (BER), nucleotide excision repair, cross-link repair and double-stranded DNA break (DSB) repair [56,57]. DSBs are one of the most severe types of DNA damage experienced in the lifetime of a cell. To survive and maintain homeostasis, two main pathways of DSB repair are known. First, homologous recombination (HR) is often associated with error-free repair, since it uses a homologous chromosome as a template. In contrast, the second pathway, nonhomologous end-joining (NHEJ), also known as illegitimate recombination, may align a few complementary bases of “microhomology” that often leads to errors such as deletions, duplications or translocation, or to the addition of sequences from elsewhere at the break site [58,59,60]. The choice between which of these pathways is used is highly regulated and depends on the type of cell, the stage of the cell cycle [60,61] and the type of genotoxic stress.

In *Ostreococcus*, DDR mechanisms have not been directly characterized. One study achieved targeted insertion of foreign DNA by transforming constructs containing a sequence homologous to the target locus by electroporation, indicating that HR repair is functional in *O. tauri* [14]. In our study, DNA insertion occurred in regions of the genome that had no homology with the vector, indicating that insertion does not primarily occur by HR, but rather by a process akin to NHEJ. This is consistent with observations of transformation methods that similarly do not use homologous regions, such as *Agrobacterium*-mediated T-DNA insertion in plants [62]. In order to gain insight into the DDR pathways that may be utilized in *O. tauri*, we searched for genes known to be required in DDR in related and model organisms. Forward genetic experiments combined with a screen for sensitivity to mutagen treatments in *Chlamydomonas* have implicated the following genes in DDR: the *REX1* gene [63], cytosolic thioredoxin h1 (*Trxh1*) (required for BER [64] and also observed in yeast [65] and mammalian cells [66], DNA polymerase zeta, DNA polymerase theta, SAE2/COM1 endonuclease, RaD17 and ERCC1 [32]. The RaD17 homolog in *Arabidopsis* was also associated with nonhomologous DSB repair [67], as was DNA polymerase theta in *Arabidopsis* [68]. Other proteins known to play a role in NHEJ repair include the Ku70/Ku80 heterodimer and DNA ligase IV in plants [69,70] and in yeast [71,72]. We confirmed by homology searches on the comparative genomics platform Pico-PLAZA3 [73] that *O. tauri* encodes four of the above-mentioned plant and algal gene families that could mediate NHEJ: the RaD17 complex (Genbank accession: OUS48190.1), which shares 20% amino-acid identity with *Arabidopsis thaliana*; DNA polymerase theta (OUS43494.1); DNA ligase IV (OUS46583.1); and the Ku70/Ku80 heterodimer (OUS47173.1).

PEG is thought to enable transformation by modification of membrane fluidity and the aggregation of phospholipid vesicles [74], as well as enhancing transformation by altering transcriptional and metabolic response in *Saccharomyces cerevisiae* [75]. Although high molecular weight PEG at high concentrations was found to reduce growth of *O. tauri* cells [15], to our knowledge, it is not mutagenic. In animals or plants, it has been shown to be completely safe to use, even for medical applications in humans [76], whereas in plants it has been used for transformation and regeneration of healthy individuals [77]. Although the exact mechanism by which foreign DNA is incorporated into the genome during PEG treatment in *O. tauri* remains to be elucidated, the combination of short- and long-read sequencing in this study has shed light on the genomic events involved. Our study suggests that DNA integration mediated by PEG occurred randomly, as none of our transformed lines shared the same integration site. This observation of random insertion is consistent with previous work [15] in which vector integration occurred in different chromosomes in 11 clonal transformants. In addition, vector insertions were accompanied frequently by structural variations at the insertion site. We found that most of the insertion events of nonvector DNA observed may have been produced by *de novo* synthesis from the cell’s recombination/repair systems, since sequences large enough to be tested by BLAST (from 55 bp to 83 bp) bore no similarities to those in common public databases. Taken together, we propose that integration of foreign DNA in *O. tauri* occurs primarily via a mechanism similar to NHEJ to repair spontaneous damage arising during cell growth.

### 4.2. Does the Integration of the Inserted DNA Have an Impact on the Deregulation of Gene Expression?

Since the genome of *O. tauri* is very compact, with only about 8000 genes and short intergenic regions [12], and these cells are haploid with few gene duplications, it is likely that a large proportion of insertion events will have been lethal and cannot be characterized using this approach. However, we easily produced thousands of transformants, demonstrating that many insertions are clearly viable, and transformation may be used to introduce DNA in different genomic locations. The integration of foreign DNA using PEG-mediated transformation seemed to occur randomly in the host genome, mainly as a single or low copy. We showed the vector may reside in coding regions, such as in the T3 line, where integration was in the coding region of the *MetAP2* gene, perhaps leading to silencing, or altered function in the interrupted gene or altered expression in adjacent or disrupted genes. Therefore, this method of PEG-mediated transformation holds promise for generating collections of insertional mutants. In particular, nonessential genes, such as those that may be induced by attack of certain pathogens or by exposure to specific environments, may be disrupted with little or no apparent effect on cell growth in culture.

In the context of this work, we performed PEG-mediated random insertion as a possible approach to answer our initial biological question, which was to elucidate the molecular basis of resistance to prasinoviruses, known to exist from previous work in *O. tauri* [52], by interrupting the genes potentially involved in this antiviral immunity system. For this reason, the choice of the host strain was based on a previous phenotypic screen for viral infection in 13 wild-type strains of *O. tauri* against 40 prasinoviruses [41]. We chose *O. tauri* RCC1115 since its genome was completely sequenced [48] and it showed a range of susceptibility to different strains of prasinoviruses. The choice of candidate transformants in this study was based on phenotypic screening for susceptibility to a panel of viruses, which initially indicated these lines had altered patterns of viral susceptibility (Figure S3). However, we found that the phenotype of resistance or susceptibility to viruses in clonal lines of RCC1115 was too unstable for use in the longer culture periods required for regrowth of strains (Figure S3). In addition, more detailed observations carried out on host-cell dynamics following viral infection (viral strain OtV09-578) revealed rapid fluctuations in growth in liquid culture (Figure S4). Importantly, we also showed that growth of transformants that were not virus-infected was not substantially affected (Figure S4), indicating that the vector insertion at these loci did not impact cell growth.

An experimental evolution experiment in the related alga *O. mediterraneus* showed that independent clonal culture lines started from single cells switched between resistant and susceptible phenotypes and this was linked to changes in size of the “small outlier chromosome” (SOC) [53]. Similarly, rapid changes in SOC size have been observed upon acquisition of resistance to prasinoviruses in *O. tauri* strain RCC4221 [78], and these resistant lines also contained a minority population of virus-susceptible cells [52]. These works are consistent with our observation of instability in the resistance phenotype in *O. tauri* RCC1115. Furthermore, it suggests that if antiviral immunity depends on the rapid generation of hypervariability in the SOC in the genus *Ostreococcus*, clones of RCC1115 would similarly display changes in SOC size. Indeed, an analysis of the karyotypes of seven transformed and three untransformed clonal lines of RCC1115 revealed genomic plasticity between all the lines independent of the transformation (data not shown); we could exclude the hypothesis that variations in chromosome size were caused by the PEG treatment, since the control (NT) lines were also affected. Moreover, the NGS data provide a clear description of rearrangements occurring in chromosome 19 leading to a large deletion of about 100 kb in line T14. Although we cannot go further in the characterization of the phenotypic effects correlated to each insertion event in the transformed lines, we could exclude a direct link between the vector insertion and the phenotypic instability of viral resistance/susceptibility during viral infection, which was better explained by a distinct mechanism of genomic plasticity.

### 4.3. PEG-Mediated Transformation as a Robust and Powerful Biomolecular Tool

To obtain a high transformation efficiency, optimal conditions were chosen according to Sanchez et al. [15]. We similarly achieved high transformation efficiency in our experiment (1.84 × 10^5^ transformed cells/µg of DNA), showing that PEG-mediated transformation was robust and reproducible. Even with high transformation efficiency, we found that DNA insertion by PEG in *O. tauri* usually led to the stable integration of a single copy per clone in the cells’ genome. Single or low copy numbers of inserts were also reported using an *Agrobacterium*-mediated transformation method in plants [38,79] and in *Clamydomonas reinhardtii* algae [80]. Having single-vector insertion events will facilitate future work aiming to characterize insertional mutants of a specific gene of interest or the effects of introducing an exogenous gene. Specifically, we showed that stable expression of the luciferase reporter introduced by PEG-mediated transformation was due to integration of the vector DNA and not transient expression, which lays the foundation for introduction of other exogenous genes of interest. Furthermore, we also showed lack of reporter-gene expression was most likely due to spontaneous deletion of the reporter gene during DNA integration, rather than gene silencing in *O. tauri*, which is in keeping with the paucity of heterochromatin in this organism [81]. Our genomic analyses also brought to light the existence of frequent indels around the vector-insertion site, as well as in the vector, which should be taken into consideration, especially when aiming to precisely insert a sequence for expression of a foreign gene. Based on all of this new knowledge about PEG-mediated transformation, we envisage using this approach as a robust and powerful biomolecular tool to go further in the study of various functional mechanisms, which has particular promise in biotechnological applications. Engineering of oleaginous algae to enhance their biomass and the bioactivity of lipid production was reported in *Nannochloropsis* sp. [82] and *Chlamydomonas reinhardtii* [83], and also in *Chlorella pyrenoidosa* for the production of biofuel and bioplastic [84]. Recently, a study showed that *Ostreococcus tauri* produced and secreted high-lipid-content droplets under conditions close to standard laboratory conditions [85], highlighting its potential as a marine algal model. Another study showed some early indications that *O. tauri* extracts were protective against cytotoxic effects of polyaromatic hydrocarbons in human cell lines [86], further indicating a potential avenue of development in bioactive compounds’ production. We propose that PEG-mediated transformation could be used as a molecular genetic approach to determine the genetic basis for a phenotype or also as a biotechnological tool. *Ostreococcus tauri* is a representative species of the order Mamiellales, including the genera *Ostreococcus*, *Bathycoccus* and *Micromonas*, which are distributed worldwide in our oceans [87,88]. The population densities of these species in different oceanic regions are considered to be indicators of climate change and nutrient availability [88,89,90], and will be important for monitoring and modeling the evolution of planetary ecosystem health and biodiversity.

## Figures and Tables

**Table 1:** Illumina sequencing and mapping statistics of reads from clonal lines against the *O. tauri* RCC1115 reference genome for untransformed (NTx) and transformed (Tx) clones. Total raw reads correspond to mapped and unmapped read pairs. The total length of the reference genome is 14,762,682 bp.

Clones	Total Raw Reads	Total Lengths of the Mapped Reads (bp)	Average Genome Coverage * (×)	Read Pairs Mapped	Proper Pairs Mapped	Mapped Reads ** (%)	Average Insert Size
NT1	90,144,988	9,104,643,788	617	62,018,612	61,681,022	68.8	249.7
NT10	98,800,514	9,978,851,914	676	75,700,644	75,183,524	76.6	275.3
T3	122,387,308	12,361,118,108	837	112,843,908	111,677,142	92.2	267.5
T6	75,540,256	7,629,565,856	517	70,533,906	69,873,506	93.4	265.7
T12	86,367,696	8,723,137,296	591	66,928,772	66,494,262	77.5	250.1
T14	79,501,816	8,029,683,416	544	66,065,344	65,680,576	83.1	260.7
T16	100,660,452	10,166,705,652	688	85,058,636	84,481,042	84.5	277.6

* Total lengths of the mapped reads/total reference genome length. ** (Read pairs mapped/raw total reads) × 100.

**Table 2:** Mapping summary of short reads back to vector sequence (pOLK4) and estimated insert copy number in each transformed (Tx) clonal line. The vector-selectable marker encodes resistance to the antibiotic G418 (809 bp). The housekeeping gene is the DNA directed DNA polymerase II gene (BE221DRAFT_187986) (3527 bp), present in a single copy in the reference genome.

Clone	Sequence Feature	Total Lengths of the Mapped Reads (bp)	Average Coverage* (×)	Average Copy Number **
T3	Vector G418 gene	680,437	841	1.01
	Housekeeping gene	2,944,554	835	
T6	Vector G418 gene	441,673	546	1.06
	Housekeeping gene	1,813,960	514	
T12	Vector G418 gene	1,833,049	2266	4.68
	Housekeeping gene	1,707,102	484	
T14	Vector G418 gene	389,557	482	0.99
	Housekeeping gene	1,718,919	487	
T16	Vector G418 gene	512,979	634	1.04
	Housekeeping gene	2,143,927	608	

* Total lengths of the mapped reads/total gene-sequence length. **Average coverage of vector G418 gene/average coverage of housekeeping gene.

## Data Availability

Publicly available datasets were analyzed in this study. The Illumina-ONT hybrid assemblies and raw Illumina and ONT reads can be found from the European Nucleotide Archive (ENA) under project accession number PRJEB43294. The sequence of the pOLK4 vector is available under GenBank accession number MW598458.

## Supplementary Materials

The following are available online at https://www.mdpi.com/2073-4409/10/3/664/s1. Figure S1: Heatmap of short reads from untransformed (NTx) and transformed (Tx) lines mapped to chromosome 19 of the reference *O. tauri* RCC1115 genome (369,517 bp), showing the large deleted region in the T14 clonal line. Note the coverage of short reads in the deleted region was not uniformly zero, as the chromosome contains many intrachromosomal repeated sequences that are expected to recruit short reads. Figure S2: Nucleotide dot-plot analyses. Alignments of the contigs from the hybrid MaSuRCA assembly in which the insertion events occurred (*y*-axis) against the reference genome and against the pOLK4 vector sequence (*x*-axis) for each transformant (**A**) T3, (**B**) T6, (**C**) T12, (**D**) T14, (**E**) T16. The length of the assembled contigs and the reference are found in Table S5. Black arrows indicate the insertion site. Red lines correspond to alignments in the same sense as the reference chromosome sequence and blue lines to antisense alignments. Figure S3: Spectrum of viral infectivity of untransformed (NTx) and transformed (Tx) clonal lines to 20 prasinoviruses (*viruses from Clerissi et al. 2012). Two plaque assay tests were carried out three months apart (Test 1 and Test 2). Dark yellow background: large difference in viral lysis phenotype in test 2 compared to test 1 result. Light yellow background: moderate difference in viral lysis phenotype in test 2 compared to test 1 result. −: no lysis; +: high lysis; +/−: low lysis. Figure S4: Cell dynamics of transformed clonal lines during 12 days postinoculation (DPI). Solid lines represent the mock-inoculated control cultures and dashed lines are cultures infected with the OtV09-578 virus (isolated in Clerissi et al. 2012) at multiplicity of infection: MOI 5. Each point represents one replicate of each culture. The peak in cell lysis occurs at 3 DPI in the T12, T14 and T16 transformed lines, in contrast to the T3 and T6 lines, where the peak in lysis is observed after 7 DPI. Note that regrowth of OtV09-578-resistant cells was observed by flow cytometry. Figure S5: Confirmation of flanking regions using PCR amplification and analysis of PCR products on electrophoresis gels. T3 line on chromosome 1 (1) at the 5′-end and (4) at the 3′-end. T6 line on chromosome 9 (3) at the 5′-end and (2) the 3′-end. T16 line on chromosome 20 (6) at the 5′-end and (5) at the 3′-end. The primers used and amplicon product size are found in Figure S7. The sequences of the PCR-products are detailed in Figure S6. CT corresponds to negative control; L: ladder (promega, G5711). Figure S6: PCR-product sequences corresponding to both flanking regions (5′-end and 3′-end) of the pOLK4 vector (dark blue) integration site in the (**A**) T3, (**B**) T6 and (**C**) T16 clonal lines. The insertions of foreign DNA of nonvector origin are shown in red. The light blue represents the duplicated region of the vector. Figure S7: Primers used in this study to confirm the flanking junctions in the T3, T6 and T16 lines. Table S1: Genomic coverage (X) of each chromosome in transformed (Tx) and untransformed (NTx) clonal lines in the *O. tauri* RCC1115 strain. The ID of the assembled contig of the reference genome in Genbank (SequenceIDs) was associated to the number of the corresponding chromosome (Chromosome) used in this study. Red color shows the corresponding reference contig where the insertion event occurred in each clonal line described in this study. Table S2: Summary of Illumina read mapping against the pOLK4 vector sequence. Total read length represents the number of processed bases from reads of total length complete vector sequence (6014 bp). Table S3: Prediction of the function of deleted unique genes on chromosome 19 in the T14 clonal line. Table S4: Raw relative luminescence units (RLU) data in untransformed (NTx) and transformed (Tx) and some thawed (D) in independent clonal lines. Table S5: Correspondence of the reference genome RCC1115 to the Masurca–Flye assembly in the transformed clonal lines.
