# Supplementary material for: Combining Nanopore and Illumina Sequencing Permits Detailed Analysis of Insertion Mutations and Structural Variations Produced by PEG-Mediated Transformation in Ostreococcus tauri
Source: Cells. 2021 Mar 17;10(3):664. doi: 10.3390/cells10030664 (PMC8002553; doi:10.3390/cells10030664)
Supplement: Supplementary file 1 [file cells-10-00664-s001.zip › Sup v1/Figure_S1.pdf]

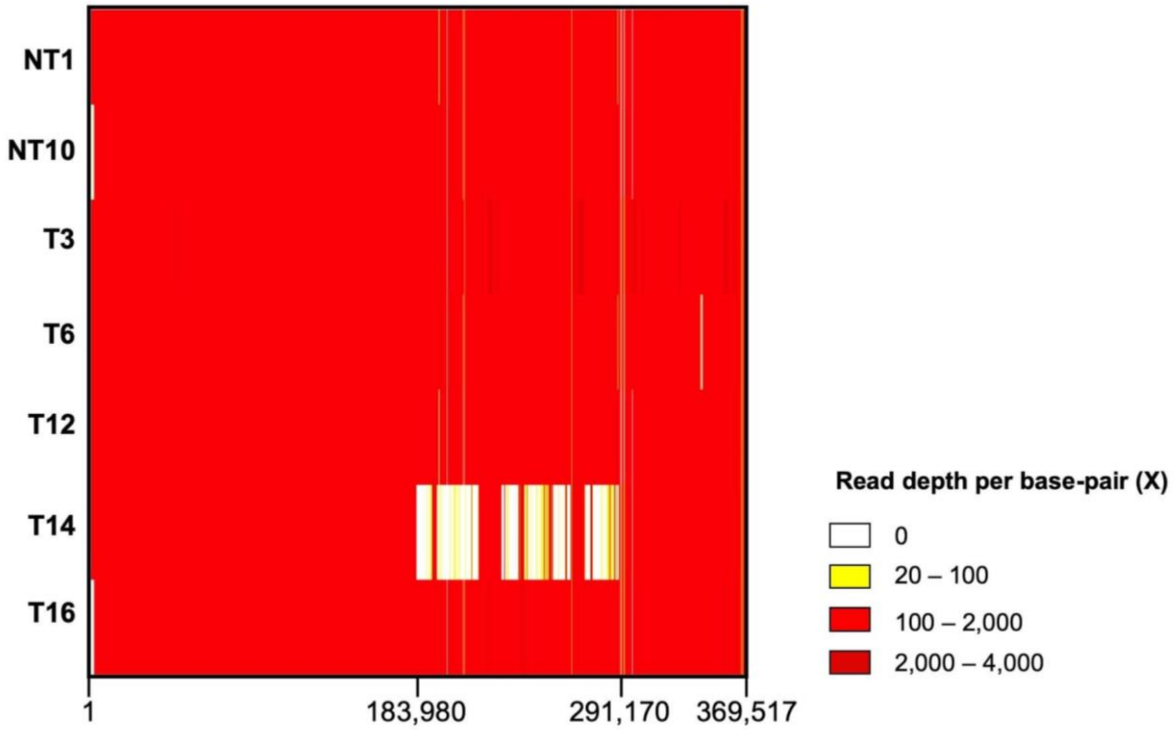

Figure S1. Heatmap of short reads from untransformed (NTx) and transformed (Tx) lines mapped to chromosome 19 of the reference *O. tauri* RCC1115 genome (369,517 bp), showing the large deleted region in the T14 clonal line. Note the coverage of short reads in the deleted region was not uniformly zero as the chromosome contains many intrachromosomal repeated sequences that are expected to recruit short reads.
