# Supplementary material for: Combining Nanopore and Illumina Sequencing Permits Detailed Analysis of Insertion Mutations and Structural Variations Produced by PEG-Mediated Transformation in Ostreococcus tauri
Source: Cells. 2021 Mar 17;10(3):664. doi: 10.3390/cells10030664 (PMC8002553; doi:10.3390/cells10030664)
Supplement: Supplementary file 1 [file cells-10-00664-s001.zip › Sup v1/Figure_S2.pdf]

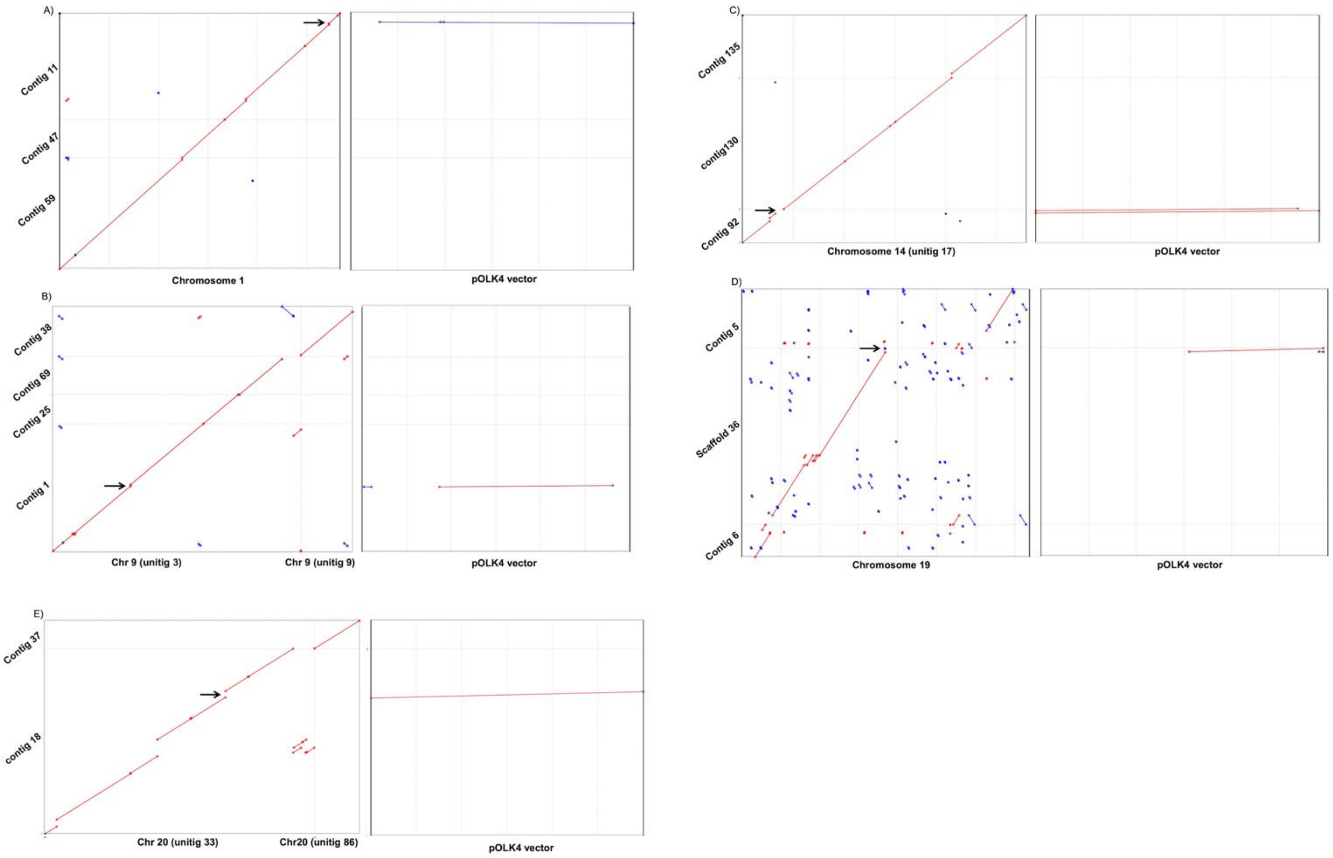

Figure S2. Nucleotide dot plot analyses. Alignments of the contigs from the hybrid MaSuRCA assembly in which the insertion events occurred (y-axis) against the reference genome and against the pOLK4 vector sequence (x-axis) for each transformant (A) T3, (B) T6, (C) T12, (D) T14, (E) T16. The length of the assembled contigs and the reference are found in Table S6. Black arrows indicate the insertion site. Red lines correspond to alignments in the same sense as the reference chromosome sequence and blue lines to antisense alignments.
