# Supplementary material for: Combining Nanopore and Illumina Sequencing Permits Detailed Analysis of Insertion Mutations and Structural Variations Produced by PEG-Mediated Transformation in Ostreococcus tauri
Source: Cells. 2021 Mar 17;10(3):664. doi: 10.3390/cells10030664 (PMC8002553; doi:10.3390/cells10030664)
Supplement: Supplementary file 1 [file cells-10-00664-s001.zip › Sup v1/Figure_S3.pdf]

Viral strain

Host strain	Lysis plaque Assay test	Virus / Clones	Sylt2-1	Sylt2-5	OtV06-1*	OtV5*	OtV09-573*	OtV06-4*	OtV09-557*	OtV09-559*	OtV19-P	OtV19-O	OtV19-T	OtV19-R	OtV09-556*	OtV09-584*	OtV09-570*	OtV06-12*	OtV09-582*	OtV09-585*	OtV09-565*	OtV09-578*
Host strain	Test 1	NT1	-	-	-	-	-	-	-	-	-	-	-	-	+/-	+/-	+/-	+	+	+	+	+
	Test 2	NT1	-	-	-	-	-	-	-	-	+/-	-	+/-	-	-	-	-	+	-	+	+	+
	Test 1	NT10	-	-	-	-	-	-	-	-	-	-	-	-	-	+/-	-	+/-	+	+	+	+
	Test 2	NT10	-	-	-	-	-	-	-	-	-	-	-	-	-	-	-	-	+	+	+	+
	Test 1	T3	-	-	-	-	-	-	-	-	-	-	-	-	-	+/-	-	+	+	+	+/-	+/-
	Test 2	T3	-	-	-	-	-	-	-	-	+/-	-	+/-	-	-	-	-	+/-	+	+	+	+/-
	Test 1	T6	-	-	-	-	-	-	-	-	-	-	-	+/-	-	+/-	+/-	+	+	+	+	+
	Test 2	T6	-	-	-	-	-	-	-	-	+/-	-	+/-	-	-	-	-	+/-	+	+	+	+/-
	Test 1	T12	-	+	-	-	-	-	-	-	-	-	-	+/-	-	-	+	+	-	-	+	+
	Test 2	T12	-	-	-	-	-	-	-	-	+	-	+	-	-	-	+/-	+	-	-	+	+
	Test 1	T14	-	+	-	-	-	-	-	-	-	-	-	+	-	-	+	+	-	-	+	+
	Test 2	T14	-	-	-	-	-	-	-	-	+	-	+	-	-	-	-	+	-	-	+	+
	Test 1	T16	-	+	-	-	-	-	-	-	-	-	-	+	-	-	+	+	-	-	+	+
	Test 2	T16	-	-	-	-	-	-	-	-	+	-	+	-	-	-	-	+	-	-	+	+

Figure S3. Spectrum of viral infectivity of untransformed (NTx) and transformed (Tx) clonal lines to twenty prasinoviruses (*viruses from Clerissi et al, 2012). Two plaque assay tests were carried out three months apart (Test 1 and Test 2). Dark yellow background: large difference in viral lysis phenotype in test 2 compared to test 1 result. Light yellow background: moderate difference in viral lysis phenotype in test 2 compared to test 1 result. -: no lysis; +: high lysis; +/-: low lysis.
