# Supplementary material for: Combining Nanopore and Illumina Sequencing Permits Detailed Analysis of Insertion Mutations and Structural Variations Produced by PEG-Mediated Transformation in Ostreococcus tauri
Source: Cells. 2021 Mar 17;10(3):664. doi: 10.3390/cells10030664 (PMC8002553; doi:10.3390/cells10030664)
Supplement: Supplementary file 1 [file cells-10-00664-s001.zip › Sup v1/Figure_S4.pdf]

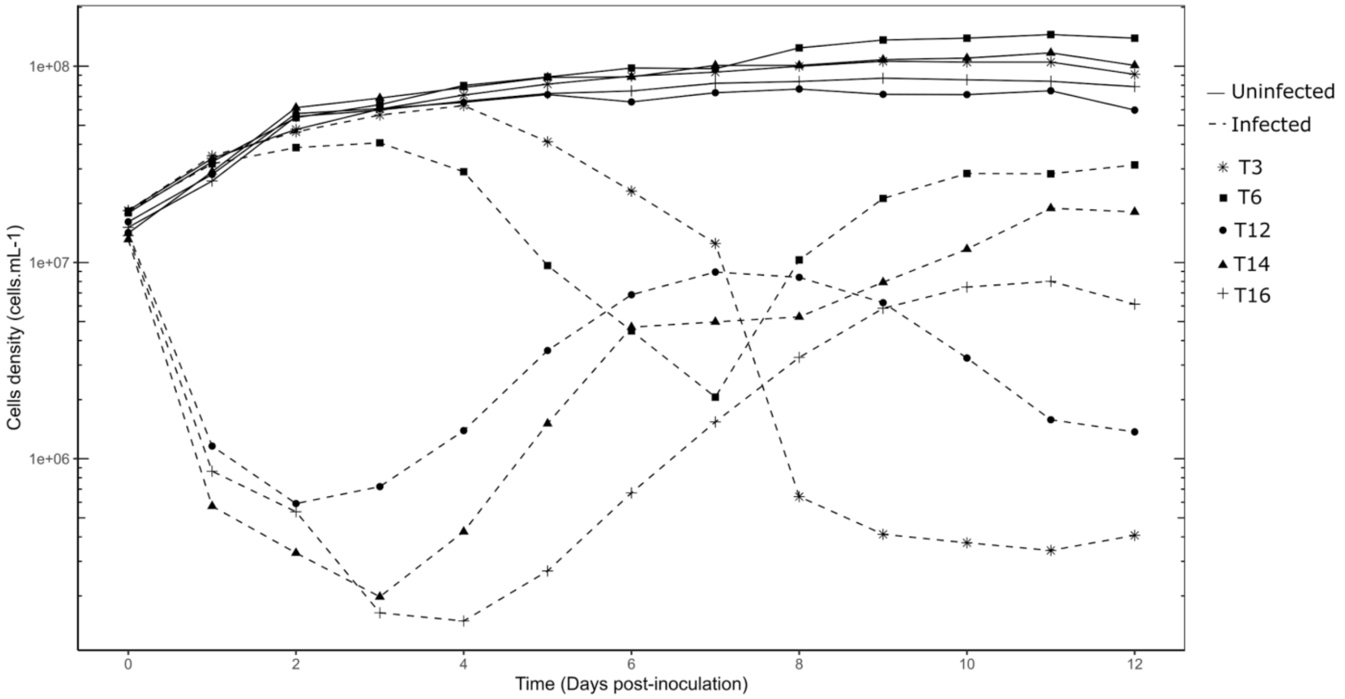

Figure S4. Cell dynamics of transformed clonal lines during 12 days post-inoculation (DPI). Solid lines represent the mock-inoculated control cultures and dashed lines are cultures infected with the OtV09-578 virus (isolated in Clerissi et al, 2012) at multiplicity of infection: MOI 5. Each point represents one replicate of each culture. The peak in cell lysis occurs at 3 DPI in the T12, T14 and T16 transformed lines in contrast to the T3 and T6 lines where the peak in lysis is observed after 7 DPI. Note that regrowth of OtV09-578-resistant cells was observed by flow cytometry.
