# Supplementary material for: Combining Nanopore and Illumina Sequencing Permits Detailed Analysis of Insertion Mutations and Structural Variations Produced by PEG-Mediated Transformation in Ostreococcus tauri
Source: Cells. 2021 Mar 17;10(3):664. doi: 10.3390/cells10030664 (PMC8002553; doi:10.3390/cells10030664)
Supplement: Supplementary file 1 [file cells-10-00664-s001.zip › Sup v1/Figure_S5.pdf]

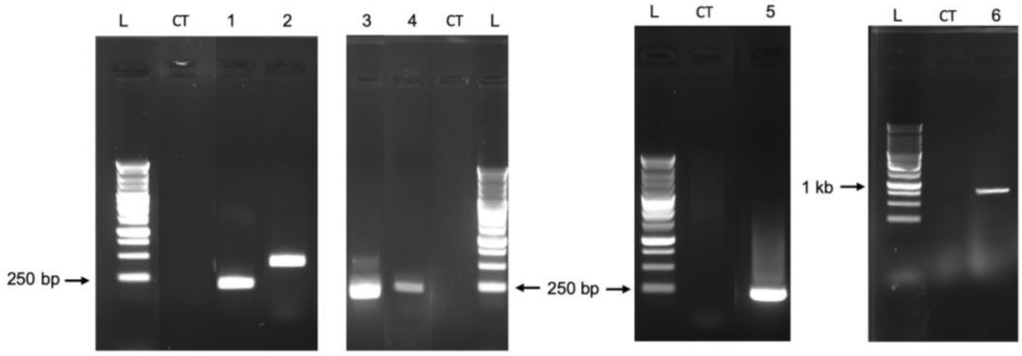

Figure S5. Confirmation of flanking regions using PCR amplification and analysis of PCR-products on electrophoresis gels. T3 line on chromosome 1 (1) at the 5′-end and (4) at the 3′-end. T6 line on chromosome 9 (3) at the 5′-end and (2) the 3′-end. T16 line on chromosome 20 (6) at the 5′-end and (5) at the 3′-end. The primers used and amplicon product size are found in the Table S5. The sequences of the PCR-products are detailed in Figure S6. CT corresponds to negative control ; L : ladder (promega, G5711).
