# Supplementary material for: Combining Nanopore and Illumina Sequencing Permits Detailed Analysis of Insertion Mutations and Structural Variations Produced by PEG-Mediated Transformation in Ostreococcus tauri
Source: Cells. 2021 Mar 17;10(3):664. doi: 10.3390/cells10030664 (PMC8002553; doi:10.3390/cells10030664)
Supplement: Supplementary file 1 [file cells-10-00664-s001.zip › Sup v1/Figure_S6.pdf]

A)

T3_5'-end of flanking junction:

5'-
TGAAGTGCTGCAGCAACTTGAACGGGCACTCCATTGATCCGTATAGGATTCACGTAATGAAT
CGGCCAACGCGCGGGGAGAGGCGGTTTGCGTATTGGGCGCTCTTCCGCTTCCTCTCAACC
AAGTCATTCTGAGAATAG-3'

T3_3'-end of flanking junction:

5'-
CGCTCAGTGGAACGAAAACTCACGTTAAGGGATTTTGGTCATGAGATTATCAAAAAGGATCT
TCACCTAGATCCTTTTTTTATACTCTGGACGGAAAACGCCCATCGACGTCACACACGCACCG
ACCATCATACTCTGGACGGAAAACGCCCATCGACGAACAACGAGGCGAGAC-3'

B)

T6_5'-end of flanking junction: 5'-
TCGCTCTCGAACTCGCGTCGCATCGACCAATGACCAATATTCCGATTGGTATGGCTTCATTC
AGCTCCGGTTCC-3'

T6_3'-end of flanking junction:
5'-
GCTGCATGTGTCAGAGGTTTTCACCGTCATCACCGAAACGCGCGAGACGAAAGGGCCTCGT
GATACGCCTATTTTTATAGGTTAATGTCATGATAATAATGGTTTCTTAGACGTCAGGTGGCAC
TTTTCGGGGAAATGTGCGCGGAACCCCTATTTGTTTATTGCGTCGATGCGACGCGAGTTATG
GGACCATTAACCGACCTCGGTGCGTTCTTGATGTTTCTGTTTCTCCGCCTGTACCCGC-3'

C)

T16_5'-end of flanking junction:
5'-
TGACCCGAGGACGCGTTCGTGGGCGACGCCGGGAGGTCTTCCTGCGCCAAGGCCGGCAG
ATCCCTGGGATCTGCGTCTCCAAAAGCGCTGGACCCTCACCAGTCACAGAAAAGCATCTTAC
GGATGGCATGACAGTAAGAGAATTATGCAGTGCTGCCATAACCATGAGTGATAACACTGCG
GCCAACTTACTTCTGACAACGATCGGAGGACCGAAGGAGCTAACCGCTTTTTTGCACAACAT
GGGGGATCATGTAACTCGCCTTGATCGTTGGGAACCGGAGCTGAATGAAGCCATACCAAAC
GACGAGCGTGACACCACGATGCCTGTAGCAATGGCAACAACGTTGCGCAAACTATTAACTG
GCGAACTACTTACTCTAGCTTCCCGGCAACAATTAATAGACTGGATGGAGGCGGATAAAGTT
GCAGGACCACTTCTGCGCTCGGCCCTTCCGGCTGGCTGGTTTATTGCTGATAAATCTGGAG
CCGGTGAGCGTGGGTCTCGCGGTATCATTGCAGCACTGGGGCCAGATGGTAAGCCCTCCC
GTATCGTAG-3'

T16_3'-end of flanking junction:
5'-
TATTATCCCGTATTGACGCCGGGCAAGAGCAACTCGGTCGCCGCATACACTATTCTCAGAAT
GACTTGGTTGAGTGGCGCGCGACGGACGACCCCCCATCTCGCGCCGCGCCACGCGT-3'

Figure S6. PCR-product sequences corresponding to both flanking regions (5'-end and 3'-end) of the pOLK4 vector (dark blue) integration site in the (**A**) T3, (**B**) T6 and (**C**) T16 clonal lines. The insertions of foreign DNA of non-vector origin are shown in red. The light blue represents the duplicated region of the vector.
