# Supplementary material for: Combining Nanopore and Illumina Sequencing Permits Detailed Analysis of Insertion Mutations and Structural Variations Produced by PEG-Mediated Transformation in Ostreococcus tauri
Source: Cells. 2021 Mar 17;10(3):664. doi: 10.3390/cells10030664 (PMC8002553; doi:10.3390/cells10030664)
Supplement: Supplementary file 1 [file cells-10-00664-s001.zip › Sup v1/Figure_S7.pdf]

Flanking junction	Target	Product size (bp)	Sequence	Chromosome	Location	Accesion number
T3-5′ end	MetAP2	221	T3F1: 5′-ACGGCAAGGAGTACCAAGTG-3′	Chromosome 1	429,218-429,237	KZ155838.1
	pOLK4		T3R1: 5′-GCTATGTGGCGCGGTATTAT-3′	Vector	5,926-5,945	/
T3-3′ end	pOLK4	320	T3F2: 5′-ACGCTCAGTGGAACGAAAAC-3′	Vector	674-693	/
	MetAP2		T3R2: 5′-ACCCACTCGGAGATGTATCG-3′	Chromosome 1	434,878-434,897	KZ155838.1
T6-5′ end	Gene unknown	186	T6F1: 5′-CTTTCGCCTTGCTTAGATCG-3′	Chromosome 9	188,626-188,607	KZ155772.1
	pOLK4		T6R1: 5′-GCGGCCAACTTACTTCTGAC-3′	Vector	87-106	/
T6-3′ end	pOLK4	415	T6F2: 5′-CGCATATGGTGCACTCTCAG-3′	Vector	5,318-5,337	/
	Gene unknown		T6R2: 5′-GAAGCGCTAAACGAGGAATG-3′	Chromosome 9	184,410-184,391	KZ155772.1
T16-5′ end	Ribosomal L19	814	T16F1: 5′-GTATTGCGATCGACGTGAAA-3	Chromosome 20	131,491-131,510	KZ155795.1
	pOLK4		T3F2: 5′-ACGCTCAGTGGAACGAAAAC-3′	Vector	674-693	/
T16-3′ end	pOLK4	239	T3R1: 5′-GCTATGTGGCGCGGTATTAT-3′	Vector	5,926-5,945	/
	Ribosomal L19		T16R2: 5′-ATGATCCTTCGTCCGTCGTC-3′	Chromosome 20	137,759-137,778	KZ155795.1

Figure S7. Primers used in this study to confirm the flanking junctions in T3, T6 and T16 lines.
