# Supplementary material for: Combining Nanopore and Illumina Sequencing Permits Detailed Analysis of Insertion Mutations and Structural Variations Produced by PEG-Mediated Transformation in Ostreococcus tauri
Source: Cells. 2021 Mar 17;10(3):664. doi: 10.3390/cells10030664 (PMC8002553; doi:10.3390/cells10030664)
Supplement: Supplementary file 1 [file cells-10-00664-s001.zip › Sup v1/Table_S2.pdf]

Clones	Total lengths of the mapped reads to vector (bp)	Average coverage (X)	Read pairs mapped to vector	Proper pairs mapped	Junction pairs mapped	Average insert size to vector
T3	3,973,340	649	39,220	35,808	1,369	284
T6	1,876,782	306	18,502	16,108	891	302
T12	11,927,090	1947	117,73	110,66	2,294	254
T14	1,276,034	208	12,604	11,418	363	335
T16	3,252,705	531	32,087	29,814	1,083	277
T3	3,973,340	649	39,220	35,808	1,369	284

Table S2. Summary Illumina reads mapping against pOLK4 vector sequence. Total reads length represents the number of processed bases from reads of total length complete vector sequence (6,014 bp).
